# Age-specificity and the evolution of senescence: a discussion

**DOI:** 10.1007/s10522-012-9410-7

**Published:** 2012-11-17

**Authors:** Maarten Wensink

**Affiliations:** 1 Max Planck Research Group: Modeling the Evolution of Aging, Max Planck Institute for Demographic Research, Konrad Zuse Strasse 1, 18057 Rostock, Germany; 2 Leyden Academy on Vitality and Ageing, Poortgebouw LUMC, Rijnsburgerweg 10, 2333 AA Leiden, The Netherlands

**Keywords:** Disposable soma, Age-specificity, Senescence, Aging, Gene expression

## Abstract

Senescence evolved because selection pressure declines with age. However, to explain senescence it does not suffice to demonstrate that selection pressure declines. It is also necessary to postulate biological mechanisms that lead to a deteriorated state of the organism at high ages, but not before. This has lead to the invocation of ‘age-specific’ genes or processes, a concept which is prone to be interpreted too freely. Events do not happen after a certain amount of time has passed. They need initiation, which means that senescence is required to be a continuous process. As a result, a change at a particular age cannot arise in isolation from changes at other ages, in particular not in isolation from changes at the ages nearby. These mechanistic constraints are not without consequence for the patterns of mortality and fecundity that can evolve. I conclude that from purely logical considerations, senescence is characterized as continuous rather than age-specific deterioration. These considerations guide (theoretical) research in the direction of investigating how continuous somatic change arises, rather than focusing at age-specific events.

## Introduction: the evolution of senescence and the meaning of age-specificity

The higher the age of an organism, the greater the organism’s contribution to fitness that cannot be affected by any event happening at that age, because that contribution lies in the past. As a result, the state of an organism at high age is under less stringent selection than the state of the organism at low age, which promotes the evolution of ‘senescence’, the deterioration of the state of an organism over ages, which negatively affects ‘vital rates’ mortality and fecundity (Medawar 1952; Williams 1957; Hamilton 1966).

To explain aging, it does not suffice to conclude that selection pressure declines over ages. It is also necessary to define the processes that are hypothesized to lead to a deteriorated state at high ages, but not before. This has lead to the invocation of ‘age-specific genes’, thus giving a genetic basis to the deteriorated state (Medawar 1952; Williams 1957; Hamilton 1966). However, this still allows for different interpretations. If ‘age-specific’ is to mean ‘a gene that is expressed at some age but not before (or after)’, there is a logical problem if such genes are taken as the source of senescence. As Kirkwood (1977) observed: “the time of action of a gene during adulthood is determined not by chronological time but by its biochemical environment”, so that the “time-keeping process” or ‘somatic change’, a change in the biochemical environment that triggers a change in gene action, should be explained before age-specific alterations in gene action can be considered. At this point it is necessary to specify what is meant by ‘gene action’. If the expression of a gene would for example lead to the accumulation of damage (see below), the rate of accumulation of damage is the gene action. The result of this action is that over time there is an increase (change) in the amount of damage that has been accumulated, while the gene action has remained unchanged. A change in the gene action itself would mean that the expression of the gene leads to a *different rate* of accumulation, which can only occur if some somatic change occurs first. Thus, there can be change of the state of the organism without a change in gene action, but there can be no change in gene action without a somatic change that initiates this change in gene action (Kirkwood and Melov 2011). Any change in gene action is *state*-*specific* rather than *age*-*specific.* This is a logical issue, unrelated to empirical evidence: events need initiation. They do not just happen because a sufficient amount of time has passed. Consequently, the process that causes senescence is necessarily continuous.

From the logical necessity that senescence is a continuous process there arises a natural alternative to the definition of age-specificity above. An ‘age-specific process’ could be defined as a process that leads to a certain state of an organism at a specific age, while actually taking place at all preceding (and subsequent) ages. The logical problem outlined above is then avoided, although it does not seem entirely correct to call such processes age-specific. From now on I refer to such processes as ‘continuous’. The question then arises whether it is possible that a continuous process has a certain effect on vital rates at some isolated age, but no effect before or after that age.

To sum up, there exist two interpretations of age-specificity: One at high risk of circularity, because in order to have age-specificity at all, it requires the existence of the very change it set out to explain, and one that avoids this risk, but for which ‘age-specificity’ may not be the correct word. While some think about senescence in terms of the latter interpretation, others have tried to formulate theories of genes, causative for senescence, that do switch expression with age, or whose expression does lead to a different outcome at different ages, while avoiding the logical problem that Kirkwood pointed out. In this paper I show that these ‘reparations’ failed, and that *if* we wish to include genes that change their action or expression at some age(s) in an evolutionary theory of senescence, such state-specific genes play a role that is qualitatively different from the role that they are currently believed to play. Furthermore I discuss the difficulties of the idea that a continuous process has a certain effect on vital rates at some isolated age, but no effect before (or after) that age. I conclude that senescence should be considered as continuous somatic change, with continuous change in vital rates.

## Age-specific deleterious effects derived from state-specific genes

Proposals to retain a place for ‘age-specific’, more correctly ‘state-specific’, genes in the evolutionary theory of senescence, appeal to (hypothetical) processes that have two characteristics. First, such processes are assumed to evolve independently of the presence of state-specific genes, so that potentially deleterious genes could measure the age of the organism from those processes. Second, such processes are postulated to have no direct effect on vital rates, so that the deleterious effect is mediated through state-specific genes, with the result that the deleterious effect takes place at some specific age. This idea is perhaps best articulated by (Dawkins 2006). He discussed a “substance S” (S for senescence) which is innocuous in itself, but which accumulates in cells, and which triggers a change of gene action when its concentration reaches a certain threshold. Thus, substance S is seen as an independent time-keeper. A similar argument from the perspective of telomere length is sometimes raised in informal discussions. A telomere is a protective DNA sequence at the end of the chromosome, the length of which is a decreasing function of age in humans (Blackburn 1991). The idea is that genes could sense the length of telomeres, and so could have age-specific effects. As discussed below, the presumed independence from state-specific genes of the somatic change cannot possibly be upheld, while the presumed innocuousness is doubtful at best.

## There is no independent time-keeping mechanism

**Fig. 1:**
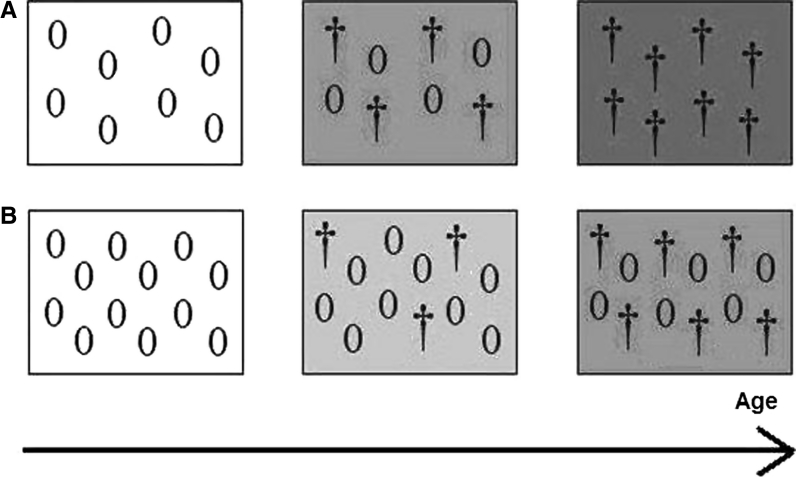
The interaction of somatic change and state-specific genes. Organisms are depicted as boxes, at different instants in time (*arrow*). State-specific genes (depicted as 0’s) can have detrimental action (when they become *daggers*), depending on the somatic change (*grey tint* of the background). The somatic change is assumed to have no noxious effect other than activating detrimental state-specific gene action. The rate of senescence is then the rate at which gene action becomes detrimental. The relevance of the somatic change to the theory becomes abundantly clear when comparing organism **a** with organism **b**. Organism **b** has a higher state-specific load (higher number of 0’s), but because of a slower somatic change, it has a lower rate of senescence

Even if substance S has no direct effect on vital rates, the triggering of deleterious age-specific genes is a far from innocuous activity. Accordingly, substance S is subject to natural selection, which means that the pace of accumulation of substance S can be manipulated by natural selection to postpone or forestall the action of potentially deleterious state-specific genes (Fig. 1). Deterioration of vital rates is caused by both substance S and the state-specific genes: Only if both factors are present does deterioration occur. The greater the number, severity, or sensitivity to somatic change of potentially deleterious state-specific genes (‘state-specific load’), the greater the deterioration that results if the somatic change triggers those state-specific genes, and the stronger natural selection will act against this somatic change. Hence, the idea of a “substance S” as a somatic change that functions as a sort of clock, independently of the presence of state-specific genes, cannot be entertained. The same goes for telomere length: Whether contributing directly to the process of senescence or not (see below), an increase in state-specific load will increase selection on the activity of telomerase [an enzyme that reverses telomere shortening (Blackburn 1991)]. This viewpoint is quite different from the idea that state-specific genes can be superimposed on some existing change, which itself evolves independently.

That somatic change does not evolve independently is demonstrated by the following. Consider again that because substance S triggers genes to exert a detrimental effect, natural selection acts on the rate at which substance S is produced and on the rate at which it is cleared. In fact, the force of selection on substance S equals the force of selection on all individual state-specific genes that are triggered by substance S added up. Clearance of substance S could come at a cost, such as in cases in which the resources (energy, metabolites) that are used to clear substance S could otherwise have been used for reproduction. Alternatively, metabolism that involves a lower rate of production of substance S could require more resources.

Now consider what happens if only one state-specific gene is present, which gives a slight increase in mortality when activated at some threshold concentration of substance S. If there is some cost of clearing substance S, this could easily outweigh the cost of the slight incrzease in mortality at some age. Now increase the state-specific load. First, the rate of senescence will increase due to the higher state-specific load. However, at some point the mortality costs could outweigh the benefits of an alternative investment of resources. Instead of removing those state-specific genes with too detrimental an effect, natural selection may lead to clearance of substance S. As a result, the detrimental effect of all state-specific genes sensitive to substance S is forestalled, and the rate of senescence decreased. Of course, other somatic changes may still make the organism deteriorate. Also, if selection pressure on the potential action of state-specific genes is low enough, benefits of preventing this action may never outweigh cost. Nevertheless, the possibility that an increase in the state-specific load leads to a lower rate of senescence is notable. Although state-specific genes certainly act as a reinforcing factor *given* a certain pace of the somatic change, following the reasoning above a higher state-specific load does not necessarily lead to a(n) (proportional) increase in the rate of senescence. This effect emerges through the evolution of a slower somatic change in response to an increase in state-specific load.

## Are there innocuous time-keeping mechanisms?

Above it was demonstrated that the ‘time-keeping’ mechanism, or somatic change, does not evolve independently of the state-specific load. In addition, we might ask whether the second putative attribute of somatic change, the lack of a direct effect on the organism’s vitality, is realistic. In the case of substance S, I suggest that it is unclear how an accumulating substance would not interfere with (cellular) signaling or the structural integrity of the organism. An effect may be expected even if some substance is chemically inactive, if only through the occupation of space, or through the addition of non-functional weight. If the somatic change is damage, as in the disposable soma theory, it is difficult to conceptualize how the damage would be free of any effect on vital rates. Indeed, central to the disposable soma theory is the idea that it is (the accumulation of) damage that leads to deterioration (Kirkwood 1977; Kirkwood and Holliday 1979; Kirkwood and Rose 1991). Similarly, changes that telomeres undergo over ages, such as loss of methylation, have been demonstrated to have direct effects on the vitality of the organism, while the shortening of telomeres with cell division is not universal (Macieira-Coelho 2011). I am not aware of any demonstration of a substance or other change that has no direct effect on vital rates, but that informs genes about the age of the organism, since Kirkwood (1977) objected along similar lines.

In conclusion there are two objections to the concept that senescence is a result of age-specific deleterious effects derived from state-specific genes. First, there is no independent time-keeping mechanism, which means that potentially deleterious state-specific genes cannot be thought of as independent from the somatic change on which their activation relies. Second, because it is doubtful whether some somatic change can be without any effect on vital rates, an underlying continuous somatic change is expected to lead to a gradual deterioration of vital rates, rather than to age-specific deterioration.

## Age-specific deleterious effects derived directly from continuous change

All the difficulties discussed above are avoided if the deterioration that characterizes senescence is viewed as the direct result of some continuous change, without mediation of some state-specific gene. With a continuous process directly causing senescence, the potential logical problem to explain what initiates deterioration does not exist. The question now arises to what extent it is possible that the deleterious effect of a continuous process takes place at some age, but not before or after. This seems to be even less likely than if the deleterious effect is mediated through a state-specific gene, in which case the somatic change is not directly harmful. As the somatic change takes place, it will likely give rise to some deterioration. There are, however, two reasons to expect that the bulk of the deleterious effect may be manifest only late. First, somatic change may be expected to be cumulative, for instance in case of cumulative damage, so that it may be expected that the higher the age, the greater the effect. Second, the amount of accumulated damage may translate into vital rates in a non-linear fashion. This could be the result of mechanisms that buffer, adapt, or remodel, leading to only a negligible decline of functioning initially (see e.g. Rattan 2006). It could also be that a decline in functioning is translated into change in vital rates in a non-linear fashion, for instance exponentially. Consequently the continuum of change has the highest effect at high ages, so that the somatic change leads to a deterioration of vital rates at high ages, but not so much before, as is required to explain senescence.

The concept of gradual somatic change rather than age-specificity is corroborated by the evidence on the mechanistic, the physiological and the demographic level. At the molecular level, there is for instance a gradual increase in molecular damage and heterogeneity (Rattan 2006, 2010), gradual malfunctioning of the cellular control systems (Hubbard et al. 2012), and decline of the integrity of mitochondrial constituents (Passarino et al. 2010). At the physiological level, senescence is characterized by gradual loss of function, for instance in the case of grip strength (Kallman et al. 1990; Andersen-Ranberg et al. 2009). At the demographic level, there is a gradual decline in fertility and a gradual increase in mortality. This is found in humans (Vaupel 2010) and in wild animals, where senescence occurs in many natural populations, and in a gradual fashion, i.e. senescence does not suddenly happen at some age (Finch 1990; Nussey et al. 2006; Jones et al. 2008; Jones et al. unpublished).

## Discussion

It is possible (but theoretically superfluous) to postulate *state*-*specific* genes on top of, but not instead of, continuous somatic change. As discussed above, the evolution of somatic change is then not independent of the state-specific genes, and some direct effect of somatic change on vital rates may be expected. If senescence is caused by an accumulation of damage that leads to a changing state of the organism, as in the disposable soma theory, this changing state could trigger potentially deleterious genes in turn. Kirkwood (1977); Kirkwood and Holliday (1979) and later Zwaan (1999) discussed this possibility, and concluded that if state-specific genes are triggered by somatic change, the process of senescence is reinforced. Certainly, cumulative damage may trigger state-specific genes. However, just as when state-specific genes are super-imposed on a substance S, an increase in the state-specific load does not necessarily increase the rate of senescence. If the accumulation of some type of damage could be prevented at low cost, an increase in the state-specific load to this damage may lead to slower accumulation of damage, with a lower rate of senescence as a result.

The conclusion that senescence is a continuous process, best characterized by decreasing deterioration over all ages, pertains to two contrasting ideas about the evolutionary theory of senescence that exist in parallel. First, there is the idea that senescence does not occur before some age at which selection pressure is virtually zero, called ‘essential lifespan’ (Rattan 2000) or ‘warranty period’ (Carnes 2011). Second, there seems to be the idea that mortality is approximately inversely related to selection pressure, although quantitative statements are not made (e.g. Partridge and Barton 1993; Charlesworth 1994). Neither of these takes seem entirely satisfactory in the context of the evolution of senescence, given the considerations in this paper. As for the concept of an ‘essential lifespan’ or a ‘warranty period’, selection pressure tends to decline in a gradual fashion. If we consider an iteroparous organism that does not senesce, the standard default situation in reasoning about the evolution of senescence, selection pressure is an exponentially declining function of age. As a result it is hard to pin down a specific lifespan that could be called ‘essential’. Only after the evolution of senescence could such an age be approximated. As selection pressure declines gradually, it would be more natural to expect senescence to be a similar gradual process, happening at all ages with deterioration approximately inverse to selection pressure, i.e. the other concept mentioned above. However, this concept is unsatisfactory because only the gradual decline of selection pressure is considered, but not the fact that the state of the organism at one age is tied to the state of the organism at preceding and subsequent ages, which takes away degrees of freedom from the patterns of senescence that can evolve. As Kirkwood and Shanley (2010) pointed out, this means that the age-pattern of selection pressure has only limited informative power, since a pattern approximately inverse to selection pressure may not be mechanistically allowed. Thus, there are two different gradual processes that interact to lead to the evolution of senescence. A straightforward way of modelling that follows from these considerations would be to trade the initial (meaning ‘at maturity’) value of the vital rates for their rate of change. An initial good performance (low mortality and/or high fecundity) then leads to faster decline (see for instance Kirkwood and Rose (1991), appendix).

To summarize, there are two requirements that the mechanisms that are believed to give rise to senescence should fulfill. First, the bulk of the deterioration should happen late rather than early. In parallel, such processes are required to be continuous rather than age-specific, for otherwise no proper account of causality is given. Processes that fulfill these conditions, conditions that are derived purely on logical grounds, are prone to be cumulative and to translate into vital rates in a non-linear fashion, so that high ages are affected much more than early ages. This does not mean that early ages are not affected at all, which may be hard to achieve from a mechanistic perspective (see above). Senescence being a gradual process, theoretical research should focus on what causes the continuous somatic change (see e.g. Kozlowski 1999; Cichon and Kozlowski 2000; Baudisch 2008; Wensink et al. 2012).

## Conclusions

1. The process that underlies senescence is one that is continuous.
2. Whether this continuous process has effects other than those mediated through state-specific genes is irrelevant to the question whether it is subject to natural selection or not. It is, and this selection should be included in theories and models.
3. State-specific genes are part of the mechanism by which somatic change affects vital rates, and they may lead to a higher rate of senescence, but also to a lower rate of senescence because of the evolution of slower somatic change in response. An increase in the rate of somatic change, on the other hand, always leads to an increase in the rate of senescence.
4. Cumulative somatic change will have some effects at early ages, although these may be negligible.
5. The main evolutionary question about senescence is what drives continuous somatic change, rather than what age-specific genes exist.
